# ILoReg: a tool for high-resolution cell population identification from single-cell RNA-seq data

**DOI:** 10.1093/bioinformatics/btaa919

**Published:** 2020-12-13

**Authors:** Johannes Smolander, Sini Junttila, Mikko S Venäläinen, Laura L Elo

**Affiliations:** Turku Bioscience Centre, University of Turku and Åbo Akademi University, Turku 20520, Finland; Turku Bioscience Centre, University of Turku and Åbo Akademi University, Turku 20520, Finland; Turku Bioscience Centre, University of Turku and Åbo Akademi University, Turku 20520, Finland; Turku Bioscience Centre, University of Turku and Åbo Akademi University, Turku 20520, Finland; Institute of Biomedicine, University of Turku, Turku, Finland

## Abstract

**Motivation:**

Single-cell RNA-seq allows researchers to identify cell populations based on unsupervised clustering of the transcriptome. However, subpopulations can have only subtle transcriptomic differences and the high dimensionality of the data makes their identification challenging.

**Results:**

We introduce ILoReg, an R package implementing a new cell population identification method that improves identification of cell populations with subtle differences through a probabilistic feature extraction step that is applied before clustering and visualization. The feature extraction is performed using a novel machine learning algorithm, called iterative clustering projection (ICP), that uses logistic regression and clustering similarity comparison to iteratively cluster data. Remarkably, ICP also manages to integrate feature selection with the clustering through L1-regularization, enabling the identification of genes that are differentially expressed between cell populations. By combining solutions of multiple ICP runs into a single consensus solution, ILoReg creates a representation that enables investigating cell populations with a high resolution. In particular, we show that the visualization of ILoReg allows segregation of immune and pancreatic cell populations in a more pronounced manner compared with current state-of-the-art methods.

**Availability and implementation:**

ILoReg is available as an R package at https://bioconductor.org/packages/ILoReg.

**Supplementary information:**

Supplementary data are available at *Bioinformatics* online.

## 1 Introduction

Single-cell RNA-seq (scRNA-seq) enables identification of known and novel cell populations by unsupervised clustering of transcriptomic profiles of individual cells. However, the high number of genes presents a major challenge for the analysis of scRNA-seq data by increasing the similarity of distances between the cells, a phenomenon known as the ‘curse of dimensionality’ (Kiselev *et al.*, 2019). To reduce its effect, scRNA-seq pipelines typically apply a feature selection step that selects a set of highly variable genes prior to unsupervised clustering (Andrews *et al.*, 2019). However, this approach can eliminate genes that are important for the identification of the underlying cell populations of a sample or add unwanted variation if irrelevant features are chosen. Moreover, the number of remaining genes is typically still in the thousands and detecting cell populations with subtle differences remains challenging.

To address this issue, we have developed a cell population identification method (ILoReg) that takes an alternative approach to dimensionality reduction by means of feature extraction. At the core of ILoReg lies a new clustering algorithm, iterative clustering projection (ICP), which transforms a gene expression matrix into a probability matrix containing probabilities of each cell belonging to k clusters. These continuous cluster probabilities provide a more practical representation of the clustering than discrete cluster labels, as they can be handled like extracted features, and they are then utilized in the consensus clustering approach that combines multiple randomly subsampled ICP solutions into a consensus solution. The consensus approach acts as a noise-reducing step prior to hierarchical clustering and visualization by non-linear dimensionality reduction, such as *t*-distributed stochastic neighbor embedding (t-SNE) (Maaten *et al.*, 2008) or uniform manifold approximation and projection (UMAP) (McInnes *et al.*, 2018). We have implemented this method as a user-friendly R package, ILoReg (https://bioconductor.org/packages/ILoReg), and demonstrate that it can greatly aid the identification of cell populations with subtle transcriptomic differences by increasing the cell population identification resolution of both clustering and visualization.

## 2 Methods and materials

### 2.1 Iterative clustering projection

**Fig. 1.**
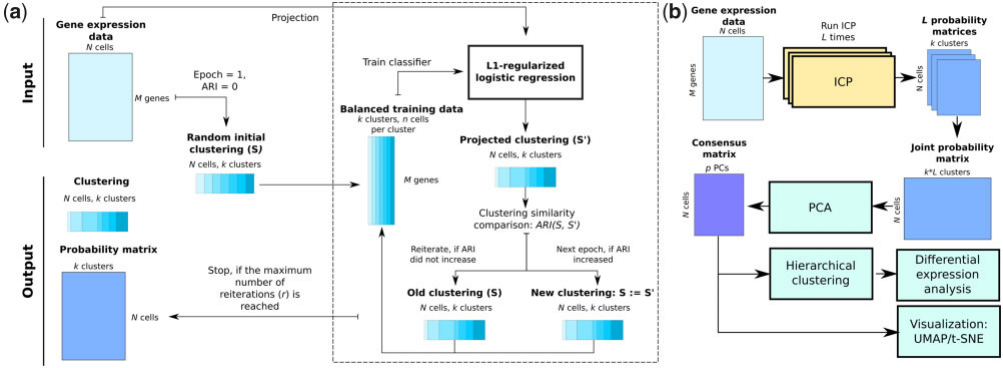
Overview of ILoReg. (**a**) Schematic of the iterative clustering projection (ICP) clustering algorithm. (**b**) Schematic of the ILoReg consensus approach for cell population identification

**Fig. 2.**
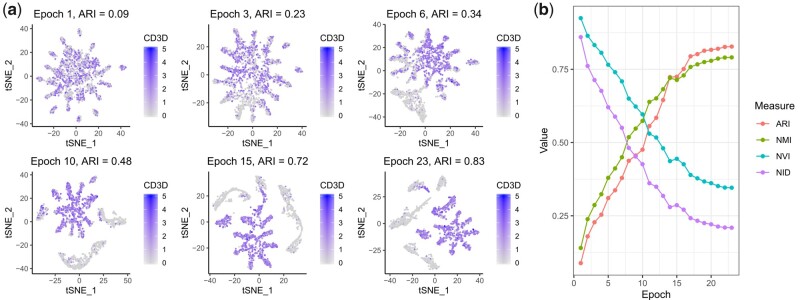
Convergence of the iterative clustering projection (ICP) clustering algorithm. (**a**) *t*-distributed stochastic neighbor embedding (t-SNE) transformations of the N×k dimensional probability matrix at different epochs of ICP, where N is the total number of cells and k is the number of clusters. The expression levels of the T cell marker gene *CD3D* are highlighted. (**b**) Clustering comparison measures calculated between the clustering and its projection at every epoch: adjusted Rand index (ARI), normalized mutual information (NMI), normalized variation information (NVI) and normalized information distance (NID). ARI at the final epoch is denoted as the projection accuracy of ICP. In this example, we used the pbmc3k dataset with k=15,  C=0.3, d=0.3 and r=5 parameter values. The clustering comparison measures in (b) were calculated using the aricode R package. See Supplementary Note S2 for an analysis on how the *d* and *C* parameters affected the projection accuracy of ICP

As a basis of ILoReg, we first introduce a new clustering algorithm, iterative clustering projection (ICP), that utilizes random down- and oversampling, supervised learning and clustering comparison to iteratively cluster the data (Figs 1a and 2). Specifically, the objective of ICP is to seek a clustering $S=\{S_{1},\ldots ,S_{k}\}$ with k clusters that maximizes the adjusted Rand index (ARI) between S and its projection S' by logistic regression:

$$\arg\underset{S}{\max\;}A\mathrm{RI}(S,S`)\;$$

In the following, we describe the four steps of the algorithm.

1. **Initialization**. Given a normalized gene expression matrix, $X={\left[x_{1},\ldots ,x_{i},\ldots ,x_{N}\right]}^{T}$, where N is the number of cells and $x_{i}$ is the transcriptional profile of the ith cell across M genes that are expressed in at least one of the N cells, the algorithm first randomly partitions the cells into k clusters, $S_{t}=\{S_{1,t},\ldots ,\;S_{k,t}\}$, in which each cluster has the same probability of being assigned to a cell, each subset contains the cell identifiers belonging to that cluster and t denotes the epoch, where t=1.
2. **Creating balanced training data**. To form a balanced training dataset $X_{t}$ and training labels $Y_{t}\;=\{Y_{1,t},\ldots ,Y_{k,t}\}$ with an equal number of cells n in each cluster of $Y_{t}$, down- and oversampling of $S_{t}$ and X are carried out. If a cluster has fewer than n cells, then its cells are oversampled with replacement. Otherwise, the cells are randomly downsampled by selecting n cells from the cluster without replacement. Since scRNA-seq datasets have very different sample sizes, n is determined by n=⌈Nd/k⌉, where d∈(0,1) and ⌈·⌉ denotes the ceiling function. Due to the sensitivity of the logistic regression model (**Step 3**) to unbalanced training data, the balancing is necessary to ensure that *k* remains unchanged during the iteration.
3. **Classifier training and projection**. An L1-regularized logistic regression classifier is trained on the training data $X_{t}$ and labels $Y_{t}$ using the LIBLINEAR library (Fan *et al.*, 2008). X is projected onto itself with the classifier, i.e. the cluster label of each of the N cells is predicted with X as input data, which yields the projected clustering ${S'}_{t}=\{{S'}_{1,t},\ldots ,\;{S'}_{k,t}\}$ and the probability matrix $P_{t}={\left[p_{1,t},\;\ldots ,\;p_{N,t}\right]}^{T}$, where $p_{i,t}$ is a real vector containing the probabilities of the *i*th cell belonging to the *k* clusters.

The objective function of the L1-regularized logistic regression model is

$$\min_{w}{\;\;\|w\|}_{1}+C\sum_{i=1}^{n}\log\left(1+e^{-y_{i}w^{T}x_{i}}\right)$$

where w is the model weight vector, n the number of training samples in a cluster, $y_{i}\in \{-1,1\}$ and ${\left\|\cdot \right\|}_{1}$ the 1-norm. The constant C > 0 determines the trade-off between regularization and correct classification, a lower value selecting fewer genes. To perform multiclass classification, the LIBLINEAR library uses the one-versus-rest scheme, in which k binary classifiers are trained using the samples belonging to one of the k clusters as positive samples and all other samples from the k-1 clusters as negatives.

**4. Clustering comparison**. The similarity between clusterings $S_{t}$ and ${S'}_{t}$ is measured by ARI (Hubert and Arabie, 1985):
  ARI=∑ijnij2-[∑iai2∑jbj2]/N212[∑iai2+∑jbj2]-[∑iai2∑jbj2]/N2

Here N is the total number of cells, $n_{ij}$ is the number of overlapping cells in clusters i and j from $S_{t}$ and ${S'}_{t}$ respectively, $a_{i}$ and $b_{j}$ are the total number of cells in clusters i and j from $S_{t}$ and ${S'}_{t}$ respectively. If ARI increases from its previous value (initialized to 0 with t=1), then $S_{t+1}$ is set to ${S'}_{t}$ and steps 2, 3 and 4 are repeated for $S_{t+1}$ in the next epoch. If ARI does not increase, the steps 2, 3 and 4 are repeated for $S_{t}$ until the maximum number of reiterations r is reached. At the start of every new epoch, the number of reiterations is set to 0. After the last reiteration, $P_{t}$ and ${S'}_{t}$ are returned as output, where t is the last epoch.

### 2.2 Consensus clustering method

**Fig. 3.**
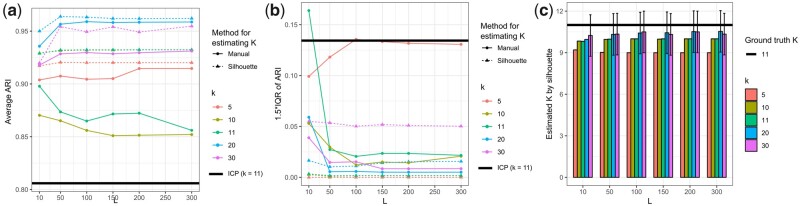
Comparison of ICP and the ILoReg consensus method. (**a**) Colored line plots showing the average adjusted Rand index (ARI) achieved by the ILoReg consensus clustering method using different values of the initial number of clusters (*k*) in iterative clustering projection (ICP) and the number of ICP runs (*L*). The average was taken over 50 different randomly initialized ILoReg consensus clustering solutions, where ARI was calculated between the inferred clustering and the reference clustering from the Pollen study. Additionally, two approaches for selecting the number of consensus clusters from the dendrogram (*K)* were compared: the silhouette method (Silhouette) and selecting the same number of clusters as in the reference clustering (Manual). The black horizontal line denotes the average ARI achieved by 500 individual ICP runs with the same number of clusters as in the reference clustering (*k *=* *11). (**b**) Line plots depicting the variability of the results in (a): 1.5* the interquartile range (IQR) of the ARI values. (**c**) The average number of estimated clusters by the silhouette method. The error bars depict 1.5*IQR of the estimated *K* from 50 different ILoReg consensus solutions. The rest of the ILoReg parameters were fixed to *C *=* *0.3, d = 0.3, *r *=* *5 and *p *=* *50

A consensus method (Fig. 1b) is used to obtain a more accurate and robust clustering of the cells than the clusterings obtained by the individual ICP runs (Fig. 3), which is also not constrained to the number of initial clusters k. The ICP algorithm is run L times with different random seeds and their probability matrices are merged to create the joint probability matrix, $P=[P_{1},\ldots ,\;P_{L}]$. The dimensionality of the data is reduced with principal component analysis (PCA) by performing eigendecomposition of the cross-product of the centered P using the RSpectra R package. Finally, the N×p dimensional consensus matrix is clustered using hierarchical clustering with the Ward’s method from the fastcluster R package (Müllner, 2013). The tree dendrogram from the hierarchical clustering is cut into K consensus clusters with the dendextend R package (Galili, 2015). The optimal number of consensus clusters can be determined automatically by the silhouette method from the cluster R package.

### 2.3 Visualization

ILoReg supports visualization using two popular non-linear dimensionality reduction methods: *t*-distributed stochastic neighbor embedding (t-SNE) from the Rtsne R package and uniform manifold approximation and projection (UMAP) from the umap R package. The N×p dimensional PCA-transformed matrix is used as input in both of the methods.

### 2.4 Benchmarking

To benchmark ILoReg against other scRNA-seq clustering methods, we considered four state-of-the-art methods: Seurat (Butler *et al.*, 2018), SC3 (Kiselev *et al.*, 2017), CIDR (Lin *et al.*, 2017) and RaceID3 (Herman *et al.*, 2018). Details of the methods are listed in Table 1. We carried out the benchmarking assuming that the true number of clusters is unknown, and therefore, used the default parameter values with each method. With RaceID3 we used the initial clustering by k-medoids without the subsequent outlier detection step that adds further clusters. With Seurat we used the number of clusters determined by the default resolution value 0.8. To measure clustering accuracy, we used ARI between the ground truth and inferred clusterings. Additionally, we compared the estimated and ground truth values of k. The related code is available online at https://github.com/elolab/iloreg-benchmarking, from which the calculating process and the parameters can be examined in more detail.

**Table 1.** Clustering algorithms used in benchmarking

	SC3	Seurat	RaceID3	ILoReg	CIDR
Clustering workflow	Feature selection + Distance matrices with three measures + PCA + k-means + CSPA + hierarchical	Feature selection + PCA + graph-based	Feature selection + k-medoids	ICP *L* times + PCA + hierarchical	Imputation + PCoA + hierarchical
Visualization workflow	None (via Scater R package)	Feature selection + PCA + t-SNE, UMAP etc.	Feature selection + t-SNE or kNN graph	ICP L times + PCA + t-SNE or UMAP	Imputation + PCoA
Method for estimating the number of clusters (*k*)	Random matrix theory	None (k by default resolution value)	Saturation	Silhouette	Calinski-Harabasz Index
Version	1.12.00	3.0.0	0.1.3	0.1.0 (Git reference ID ‘85196be6’)	0.1.5

For benchmarking we selected eleven public scRNA-seq datasets from three studies, in which each cell has been categorized by the authors of the original publication. The benchmarking datasets that we used are listed in Table 2. The Baron and Galen datasets are silver standard datasets, i.e. their clusters were identified by the authors based on gene markers. The Pollen dataset is a gold standard dataset with information on which cell line each cell originated from. Before clustering we removed spike-ins from the Pollen dataset. The normalization of each dataset was performed using the same method that was given in the original study.

**Table 2.** Summary of the datasets used in benchmarking

Study	Organism	No. of cells	No. of clusters	Protocol	Units	Standard
Pollen (Pollen *et al.*, 2014)	Human	301	11	SMARTer	TPM	Gold
Baron (Baron *et al.*, 2016)	Human	1303–3605	14	inDrop	UMI	Silver
Galen (van Galen *et al.*, 2019)	Human	108–3738	5–15	Seq-Well	UMI	Silver

### 2.5 Preprocessing of the pbmc3k dataset

We used a peripheral blood mononuclear cell (PBMC) dataset (pbmc3k) to compare the cell subsets found by the benchmarked methods. The raw FASTQ reads of the pbmc3k dataset were downloaded from the public database of the 10X Genomics company (https://support.10xgenomics.com/single-cell-gene-expression/datasets) and the preprocessing was performed using Cell Ranger v2.2.0 and the GRCh38.p12 human reference genome. The unique molecular identifier (UMI) counts were normalized using the LogNormalize method from the Seurat R package.

### 2.6 Functional analysis of the Baron1 dataset

To identify enriched biological pathways among the differentially expressed genes between the healthy and injured beta cell populations from the Baron1 dataset, we used the Metascape web tool (Zhou *et al.,* 2019) .

### 2.7 Run time and memory usage

The run time and maximal resident set size (RSS) of the five benchmarked methods were measured using two PBMC datasets: pbmc3k (∼3k cells) and a subset of the fresh_68k_pbmc_donor_a dataset (20k cells) on a cluster node with CentOS Linux 7 operating system, 12-core 2.66 GHz Intel Xeon X5650 processor and 96 GB 1066 MHz DDR3 of RAM. The workflow steps that were included in this comparison were dimensionality reduction, clustering and estimating the optimal number of clusters. In contrast to the other methods, changing the number of clusters k with SC3 can be time-consuming due to the computational bottleneck step involving *k*-means clustering. Since in practice the user needs to run the consensus clustering with a range of different k values, we adjusted the SC3 workflow to use k values ranging from 2 to 50. 12 threads were used with the methods that support parallel computing (SC3 and ILoReg).

### 2.8 Differential expression analysis

The ILoReg R package provides user-friendly functions that enable identification of gene markers for clusters and visualization of gene expression across cells and between clusters (Supplementary Note S1). The current implementation of ILoReg supports two functions for gene marker identification. The first function, *FindAllGeneMarkers*, allows simultaneous identification of gene markers for all K clusters. Differential expression analysis is performed using the one-versus-rest scheme, in which cells from each cluster are compared against the rest of the cells. To accelerate the analysis, the user can apply filters to remove genes that are less likely to be good marker genes or downsample cells. The differential expression analysis uses the Wilcoxon rank-sum test to calculate a *P*-value representing the statistical significance of a gene. The *P*-value adjustment for multiple comparisons is carried out using the Bonferroni method. A second function, *FindGeneMarkers*, enables comparison between any two arbitrary sets of clusters.

## 3 Results

### 3.1 Benchmarking

**Fig. 4.**
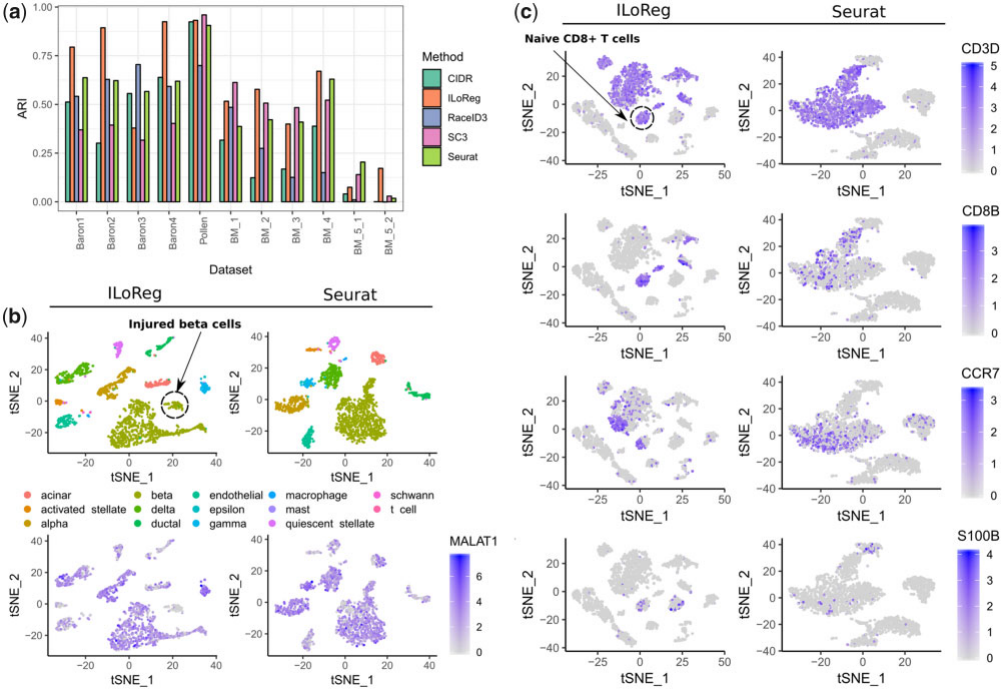
Benchmarking and identification of cell populations with subtle transcriptomic differences. (**a**) Benchmarking of ILoReg against four other scRNA-seq clustering methods: Seurat, SC3, CIDR and RaceID3. To compare the methods, the adjusted Rand index (ARI) was calculated in eleven datasets between the inferred clustering of each method and the reference clustering from the original study. The respective cluster number estimates are provided in Supplementary Figure S1. The datasets with the ‘BM’ prefix are from the van Galen study. (**b**) Comparison of *t*-distributed stochastic neighbor embedding (t-SNE) plots generated by ILoReg and Seurat for the Baron1 dataset, highlighting the expression levels of *MALAT1* that differentiates injured from healthy beta cells. The cells were colored by the reference labels of the original study. (**c**) Comparison of t-SNE plots generated by ILoReg and Seurat for the pbmc3k dataset, highlighting the expression levels of four genes (*CD3D*, *CD8B*, *CCR7*, *S100B*) that distinguish naive CD8+ T cells. All the analyses were carried out using the default parameter values of Seurat and ILoReg (*k = 15, C = 0.3, d = 0.3, r = 5, p = 50, L = 200*)

We benchmarked ILoReg against four other clustering methods (Butler *et al.*, 2018; Herman *et al.*, 2018; Kiselev *et al.*, 2017; Lin *et al.*, 2017), Seurat, SC3, CIDR and RaceID3, each functioning on a largely different principle (Table 1), using eleven gold (Pollen) or silver (Baron and van Galen data) standard datasets from three publicly available studies (Baron *et al.*, 2016; van Galen *et al.*, 2019; Pollen *et al.*, 2014) (Table 2). Although in many cases the numbers of clusters estimated by the algorithms were close to what the authors of the original studies reported (Supplementary Fig. S1), comparison by ARI between the inferred and original clusterings revealed considerable inaccuracies (Fig. 4a). ILoReg performed generally well regardless of the sample size, whereas CIDR and RaceID3 performed worse on average. In contrast to the other methods, SC3 tended to overestimate the number of clusters for larger datasets (e.g. Baron) and it was more accurate with smaller datasets (e.g. Pollen). Seurat performed consistently across the datasets, but for most datasets worse than ILoReg. In two of the datasets from the van Galen study (BM5-34p and BM5-34p38n), none of the methods were able to achieve even moderate accuracy for them. These datasets were sorted by flow cytometry and had highly unbalanced cluster labels in the original study, whereas in our comparison the inferred clusterings were more uniformly distributed, explaining the discrepancy.

### 3.2 Segregation of healthy and injured pancreatic beta cells

From the Baron1 dataset ILoReg identified a subpopulation of pancreatic beta cells (Fig. 4b) with *MALAT1* downregulated (Wilcoxon rank-sum test, adjusted *P *<* *0.01, log2 FC ∼ -1.5). *MALAT1* is a gene that inhibits apoptosis and has been found to be negatively correlated with post-isolation islet cell death (Wong *et al.*, 2019). In line with this, functional analysis of the differentially expressed genes between the two beta cell populations revealed a process that has been previously linked to beta cell destruction, i.e. endoplasmic reticulum stress (Supplementary File S1), further indicating the cluster indeed comprises injured beta cells (Eizirik *et al.*, 2008). By contrast, the beta cells in the t-SNE representation of Seurat distributed more densely and there was no clear distinction between the *MALAT1*- and *MALAT1*+ cell populations (Fig. 4b).

### 3.3 Identification of peripheral blood mononuclear cell subsets

**Fig. 5.**
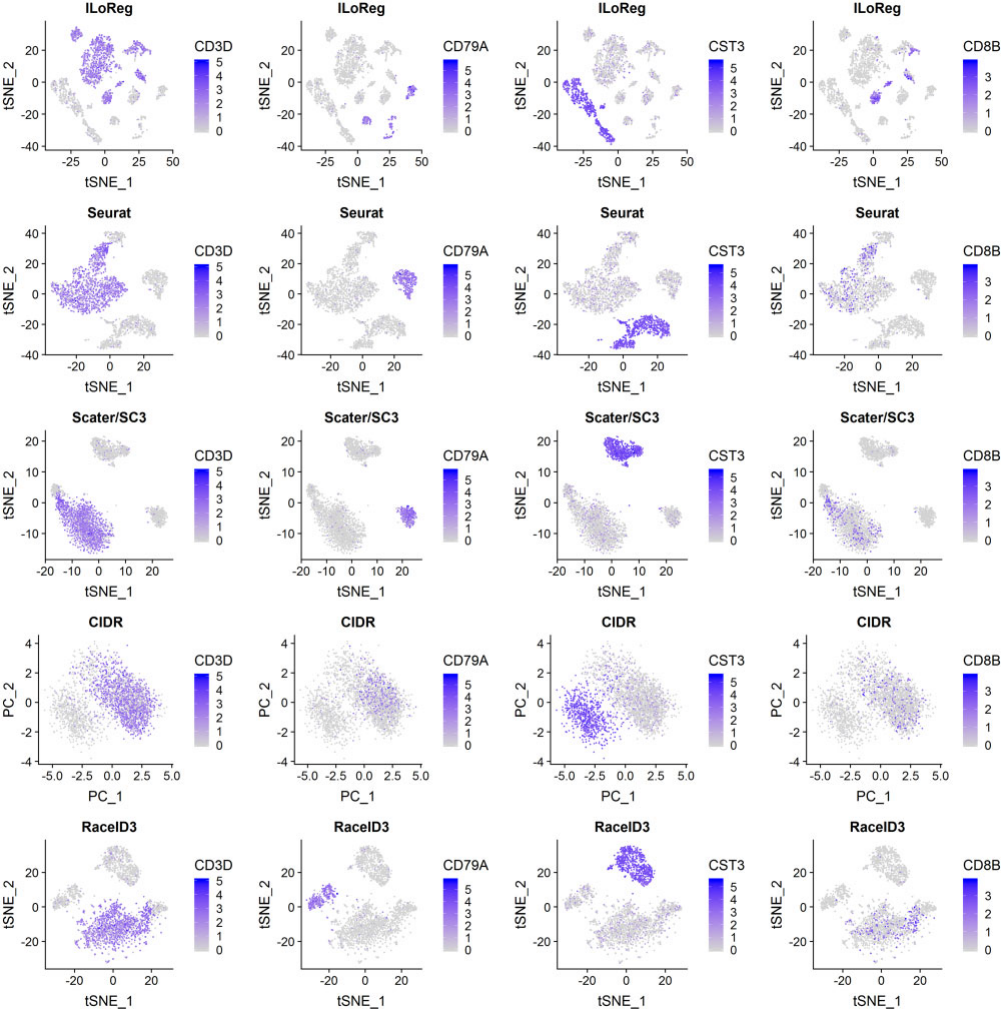
Visualization comparison between the five benchmarked methods in the pbmc3k dataset. Expression levels of gene markers for major peripheral blood mononuclear cell (PBMC) types are highlighted over two-dimensional visualizations of each benchmarked method: *CD3D* (a T cell marker), *CD79A* (a B cell marker), *CST3* (a marker for monocytes, dendritic cells and platelets) and *CD8B* (a CD8+ T cell marker). SC3 is the only method without its own two-dimensional visualization function, but the scater R package (McCarthy *et al.*, 2017) was used to perform the visualization, as recommended in the manual of the SC3 R package

In the pbmc3k dataset, a comparison between the five benchmarked methods (Fig. 5) showed that the two-dimensional visualizations of Seurat, SC3 and RaceID3 were similar, containing three main clusters: (i) T cells and NK cells; (ii) dendritic cells and monocytes and (iii) B cells. With CIDR, the B and T cells were overlapping in the visualization, suggesting its representation to be least optimal among the five methods. On the contrary, multiple distinct subpopulations were clearly visible within each main cell type in the t-SNE representation of ILoReg. Interestingly, unlike the other methods, ILoReg identified a cluster that expressed *CD3D, CD8B, CCR7* and *S100B* genes (Fig. 4c); corresponding to naive CD8+ T cells (Oetjen *et al.*, 2018). Overall, based on the expression of *CD8B* the segregation of CD8- and CD8+ T cells by ILoReg was clearly most accurate. A more comprehensive analysis (Supplementary Note S1) revealed further cell populations that are in agreement with past studies, such as CD56+ and CD56++ NK cells (Stoeckius *et al.*, 2017), naive and memory B cells with lambda or kappa light chain (Agematsu *et al.*, 2000, 27; Giachino *et al.*, 1995; Monaco *et al.*, 2019), as well as rare platelets, which typically constitute less than 1% of PBMCs (Zheng *et al.*, 2017).

To investigate the inability of Seurat to identify the naïve CD8+ T cells, we first discovered that many of the important naïve CD8+ T cell markers were missing from the set of 2000 highly variable genes (HVG) selected by Seurat, *S100B* being the only gene from the visualization in Figure 4c that was present in the set. Next, we adjusted the number of principal components to rule out that the number of principal components would be causing the issue and found that the naïve CD8+ T cell population was still not identifiable in the t-SNE plots (Supplementary Fig. S2). Therefore, it is highly likely that the naïve CD8+ T cell markers have either too weak signal for PCA to form the correct principal component or too many of the relevant genes are missing from the HVG set. The analysis shows that the selection of a fixed number of highly variable can be suboptimal, preventing identification of important cell types, and that the L1-regularization based approach of ILoReg can be used to identify these cell types.

### 3.4 Adjustment of ILoReg parameters

To set default values for the parameters of ILoReg and understand their functioning better, we performed various analyses to investigate how the parameters affected the result. A full description of the investigation is included in Supplementary Note S2. ICP has in total four parameters, for which we determined the following descending order of importance: (i) the number of clusters (*k*), (ii) *d* that controls the number of cells (*n*), (iii) *C* that controls the trade-off between correct classification and regularization in the logistic regression model and (iv) the maximum number of reiterations (*r*). Both *k* and *d* were clearly important determinants of the resolution of the cell population identification. As a higher *k* leads to a higher number of predicted cell populations, this is also reflected in the consensus clustering. Interestingly, the relation between *d* and the resolution was inverse, a higher value resulting in a smaller resolution. As *C* and *r* had less effect on the results, the user should primarily tune *k* and *d* to fine-tune the clustering. As two general guidelines, increasing *d* from 0.3 to 0.4–0.7 helps if the result feels over-clustered, and testing a *k* value higher (e.g. 30) than *k *=* *15 is recommended to ascertain the full potential of ILoReg. The consensus approach has two additional parameters: the number of ICP runs (*L*) and the number of principal components (*p*). Changing *L* from its default (200) is not recommended, but tuning *p* from its default (50) with the help of the elbow plot provides a fast way to control the resolution by including a different number of uncorrelated probabilities into the consensus solution.

### 3.5 Run time and memory usage

The most computationally intensive part of ILoReg is running ICP *L* times (default *L *=* *200). To accelerate it, the R package supports computing the ICP runs in parallel. Using the default parameter values and 12 threads, the run times for ∼3000 cells and ∼20 000 cells were ∼1 and ∼10 h, respectively (Table 3). It should be noted, however, that although the run time of a single workflow was relatively long, the workflow is generally simple and the number of consensus clusters *K* is very fast to change (∼1 s with ∼3000 cells). By contrast, with SC3 the user must repeat the clustering for each number of clusters *k* separately, thus roughly multiplying the run time by the number of different *k* values. Similarly, with Seurat the user must repeat the graph-based clustering with different resolution values without knowing how many clusters a resolution value gives. Another considerable benefit of ILoReg is its ability to find subpopulations without the need for further sub-clustering, therefore considerably simplifying the analysis workflow and saving further time.

**Table 3.** Run time and memory usage

Method	Number of cells	Run time	Max. resident set size (kB)
CIDR	3000	00h05m50s	3 106 072
CIDR	20 000	10h09m22s	36 010 516
ILoReg	3000	01h15m32s	660 792
ILoReg	20 000	10h05m38s	13 916 804
RaceID3	3000	00h08m47s	3 038 620
RaceID3	20 000	37h56m31s	21 633 488
SC3	3000	05h37m04s	5 472 304
SC3	20 000	44h42m48s	29 116 792
Seurat	3000	00h00m58s	819 452
Seurat	20 000	00h04m47s	5 283 004

## 4 Discussion

While many methods have been developed for the unsupervised clustering of scRNA-seq data, one of the persisting challenges in the clustering of scRNA-seq data is the high dimensionality of the data. In scRNA-seq data the number of features is typically in tens of thousands, but many cell populations of potential interest can be differentiable by the expression of only a few genes. Therefore, the targeted biological signal is hidden in the vast amount of technical and biological noise present in the data. To mitigate the noise, dimensionality reduction through feature selection is routinely performed prior to clustering. However, this approach suffers from the consequence of selecting only a fixed number of highly variable genes, some of which can be irrelevant to the biological signal the user aims to extract, whereas others that are discarded can be relevant to it.

In this article, we have introduced a new method (ILoReg) to cell population identification that utilizes a novel unsupervised learning algorithm (ICP) that performs both clustering and feature extraction by iteratively seeking a clustering that maximizes the predictability of the clustering by supervised learning. The simultaneous feature extraction is performed through logistic regression, which provides the cluster probabilities for the clustering. Remarkably, through integration of feature selection and clustering by L1-regularization ICP manages to overcome the issue of selecting a fixed number of genes. Instead, genes are selected and weighted during every step of the iteration by their relevance in predicting the current clustering, helping to assure that only such genes are selected that are differentially expressed among populations of cells. To mitigate the irreproducibility of ICP, ILoReg uses the consensus approach that runs ICP multiple times and combines their results by PCA and hierarchical clustering.

The approach introduced in this article falls into machine learning categories that have so far had relatively few applications in computational biology. As the cluster labels are not strictly defined in the probability space, ICP can be conceptually regarded as a fuzzy (soft) clustering algorithm. Moreover, one can consider ICP a self-supervised learning algorithm, because it trains a classifier using unlabeled data. To our knowledge, this is the first study to demonstrate the applicability of this type of self-supervised learning methods to downstream analysis of scRNA-seq data.

To demonstrate the ability of ILoReg to identify biologically meaningful cell subsets from scRNA-seq data, we first showed that ILoReg identified several PBMC subsets that are differentiable by the expression of only a few genes. This included naive CD8+ and CD4+ T cells, multiple effector T cell subtypes, as well as some unconventional subsets, such as B cells with lambda and kappa light chains. In our second experiment, we studied pancreatic cell subtypes using ILoReg and found *MALAT1*- beta cells, which correspond to injured beta cells. Importantly, these experiments demonstrated the superiority of ILoReg in finding rare cell subsets compared to current state-of-the-art methods (Seurat, SC3, CIDR and RaceID3). Another significant advantage of ILoReg is that it enables more accurate visualization of cells with existing non-linear dimensionality reduction methods, such as t-SNE and UMAP, because like in the clustering, the input of these methods is derived probabilistically. By first forwarding the cluster probabilities extracted by ICP to PCA, and subsequently to a non-linear dimensionality reduction method, ILoReg provided a representation that segregated the different immune and pancreas cell subsets into more distinct clusters compared to the other benchmarked methods.

The approach introduced in this article raises also new challenges. First, the run time of ILoReg was significantly higher (1 and 10 h for 3 k and 20 k cells, respectively) than for the fastest method of the comparison (Seurat), but comparable with the three other benchmarked methods (CIDR, SC3, RaceID3). Since scRNA-seq datasets are expected to continue to grow in size, further development is necessary to improve the efficiency of the algorithm. One solution could be GPU computation that has already shown great potential in computational genomics, with up to > 200-fold decreases in run times (Taylor-Weiner *et al.*, 2019). Secondly, the clustering can be sensitive to the hyperparameters of ILoReg, most importantly the number of clusters in ICP (*k*) and the *d* parameter that controls the number of cells in the training data. Since finding an optimal clustering may require tuning the hyperparameters, we have provided instructions for tuning them in the Results section. Thirdly, ICP is stochastic, which forces setting a seed for the random number generation to obtain reproducible results. However, our results suggested that the consensus method performs robustly and the effect of the initialization is generally small. Fourthly, loss of interpretation may be experienced as the components of PCA are no longer gene-derived, but instead based on the ICP cluster probabilities, preventing the use of gene-component heatmaps to determine which components to include for further downstream analysis. Finally, due to the training data balancing the clusters that ICP finds have, on average, similar size, but in real tissue-specific scRNA-seq data the cell numbers across cell types can greatly vary. However, our results showed that the ILoReg consensus method was able to identify even extremely rare cell populations, such as dendritic cells and platelets, that have a prevalence of only few percentages in PBMC tissue. More investigation is needed to determine whether the method could be improved by modifying ICP to better account for cell populations of different sizes.

In conclusion, ILoReg is a promising new method for clustering and visualization of high-dimensional data. In particular, our results demonstrate that ILoReg can greatly aid the identification of cell populations with subtle transcriptomic differences.

## Supplementary Material

btaa919_Supplementary_Data
